# *N*-(2,6-Dimethyl­anilino)-5,6-dihydro-4*H*-1,3-thia­zin-3-ium chloride monohydrate

**DOI:** 10.1107/S160053680801372X

**Published:** 2008-05-10

**Authors:** Mikelis V. Veidis, Liana Orola, Reinis Arajs

**Affiliations:** a University of Latvia, Kr. Valdemara 48, Riga, LV 1013, Latvia

## Abstract

In the title compound, alternatively called xylazine hydro­chloride monohydrate, C_12_H_17_N_2_S^+^·Cl^−^·H_2_O, the six-membered thia­zine ring is in a half-chair conformation. In the crystal structure, six component centrosymmetric clusters are formed *via* inter­molecular O—H⋯Cl, N—H⋯O and N—H⋯Cl hydrogen bonds involving xylazine cations, chloride anions and water mol­ecules.

## Related literature

For related literature see: Carpy *et al.* (1979 ▶); Kalman *et al.* (1977 ▶).
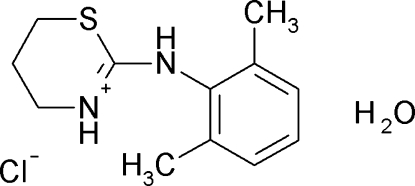


## Experimental

### 

#### Crystal data

C_12_H_17_N_2_S^+^·Cl^−^·H_2_O
*M* *_r_* = 274.81
Monoclinic, 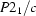

*a* = 13.4546 (2) Å
*b* = 8.6547 (1) Å
*c* = 12.7732 (2) Å
β = 109.210 (2)°
*V* = 1404.56 (4) Å^3^
*Z* = 4
Cu *K*α radiation
μ = 3.69 mm^−1^
*T* = 100 K
0.44 × 0.25 × 0.14 mm

#### Data collection

Oxford Diffraction Xcalibur diffractometer
Absorption correction: numerical (de Meulenaer & Tompa, 1965 ▶) *T*_min_ = 0.30, *T*_max_ = 0.61
19046 measured reflections
2747 independent reflections
2509 reflections with *I* > 2σ(*I*)
*R*_int_ = 0.029

#### Refinement

*R*[*F* ^2^ > 2σ(*F* ^2^)] = 0.033
*wR*(*F* ^2^) = 0.088
*S* = 1.01
2509 reflections
154 parameters
H-atom parameters constrained
Δρ_max_ = 0.43 e Å^−3^
Δρ_min_ = −0.33 e Å^−3^

### 

Data collection: *CrysAlis CCD* (Oxford Diffraction, 2007 ▶); cell refinement: *CrysAlis RED* (Oxford Diffraction, 2007 ▶); data reduction: *CrysAlis RED*; program(s) used to solve structure: *SIR92* (Altomare *et al.*, 1994 ▶); program(s) used to refine structure: *CRYSTALS* (Betteridge *et al.*, 2003 ▶); molecular graphics: *ORTEP-3 for Windows* (Farrugia, 1997 ▶); software used to prepare material for publication: *CRYSTALS*.

## Supplementary Material

Crystal structure: contains datablocks global, I. DOI: 10.1107/S160053680801372X/lh2620sup1.cif

Structure factors: contains datablocks I. DOI: 10.1107/S160053680801372X/lh2620Isup2.hkl

Additional supplementary materials:  crystallographic information; 3D view; checkCIF report

## Figures and Tables

**Table 1:** Hydrogen-bond geometry (Å, °)

*D*—H⋯*A*	*D*—H	H⋯*A*	*D*⋯*A*	*D*—H⋯*A*
N5—H5⋯O17	0.87	1.97	2.815 (2)	163
O17—H171⋯Cl16^i^	0.82	2.36	3.158 (1)	164
N7—H7⋯Cl16^i^	0.87	2.37	3.204 (1)	162
O17—H172⋯Cl16^ii^	0.83	2.35	3.171 (1)	173

Symmetry codes: (i) 
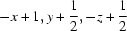
; (ii) 
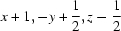
.

**Fig. 1.**
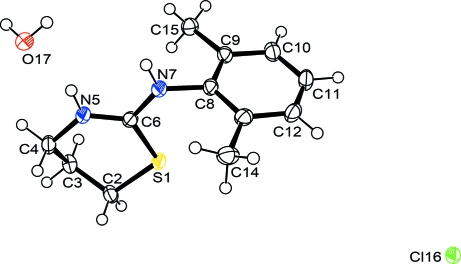
The molecular structure of the title compound with thermal ellipsoids drawn at the 50% probability level.

**Fig. 2.**
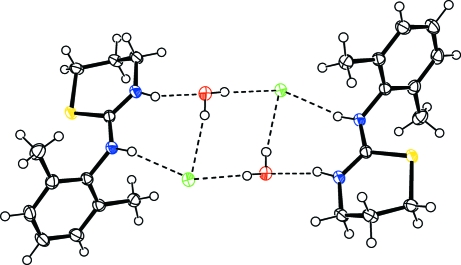
Intermolecular hydrogen bond formation (dashed lines) in the title compound.

## supplementary crystallographic information

### Comment

Xylazine hydrochloride monohydrate is a pharmaceutical used in veterinary medicine as an anesthetic. The substance is an alpha2-agonist with sedative, analgesic, and muscle relaxing properties.

The crystal structure of the title compound has been determined at 100 K. The structure is depicted in Fig. 1. The phenyl ring forms a dihedral angle of 83.24 (14)° with the plane defined by S1, C6 and N5 of the thiazine ring. The six-member thiazine ring assumes the half-chair conformation.

Hydrogen atoms are bonded to both nitrogen atoms forming a cation. Both hydrogen atoms participate in hydrogen bonding. The two xylazine moieties are held together through an extended H-bond network involving the nitrogen, oxygen, and chlorine anions. In the crystal structure, centrosymmetric clusters are formed by N—H···O—H···Cl···H—N hydrogen bond sequence between the two xylazine moieties.

There are H-bonds which do not join the xylazine moities between oxygen and chlorine (Fig. 2). These may impart additional rigidity in the cluster. As a result of Cl···H—O hydrogen bonding a parallelogram is formed by the Cl—O—Cl—O atoms.

The hydrogen bond lengths are given in the Table 1.

### Experimental

The title compound was supplied by Grindeks Company. For crystal structure determination suitable crystals were grown by slow evaporation of an ethanol (96%) solution at room temperature.

### Refinement

The hydrogen atoms were located by difference Fourier method. During refinement hydrogen atoms were costrained to the riding mode. *U*_iso_(H)=*xU*_eq_(C,*N*,*O*), where the average values of *x* are 1.15 for H atoms bonded to the thiazine ring, 1.48 for methyl H atoms, 1.16 for benzene ring H atoms, 1.17 fot the H atoms bonded to the nitrogen atoms and 1.44 for the H atoms of the water molecule.

### Crystal data

**Table:**

C_12_H_17_N_2_S^+^·Cl^–^·H_2_O	*F*_000_ = 584
*M**_r_* = 274.81	*D*_x_ = 1.300 Mg m^−^^3^
Monoclinic, *P*2_1_/*c*	Cu *K*α radiation λ = 1.5418 Å
Hall symbol: -P 2ybc	Cell parameters from 19046 reflections
*a* = 13.4546 (2) Å	θ = 3.5–74.6º
*b* = 8.6547 (1) Å	µ = 3.69 mm^−^^1^
*c* = 12.7732 (2) Å	*T* = 100 K
β = 109.210 (2)º	Prism, white
*V* = 1404.56 (4) Å^3^	0.44 × 0.25 × 0.14 mm
*Z* = 4	

### Data collection

**Table:**

Oxford Diffraction Xcalibur diffractometer	2747 independent reflections
Radiation source: Enhance (Cu) X-ray Source	2509 reflections with *I* > 2.0σ(*I*)
Monochromator: graphite	*R*_int_ = 0.029
*T* = 100 K	θ_max_ = 74.6º
φ and ω scans	θ_min_ = 3.5º
Absorption correction: numerical(de Meulenaer & Tompa, 1965)	*h* = −16→16
*T*_min_ = 0.30, *T*_max_ = 0.61	*k* = −10→10
19046 measured reflections	*l* = −15→15

### Refinement

**Table:**

Refinement on *F*^2^	H-atom parameters constrained
Least-squares matrix: full	W = [*w*eight][1-(δ*F*/6σ*F*)^2^]^2^
*R*[*F*^2^ > 2σ(*F*^2^)] = 0.033	(Δ/σ)_max_ = 0.0003
*wR*(*F*^2^) = 0.088	Δρ_max_ = 0.43 e Å^−^^3^
*S* = 1.02	Δρ_min_ = −0.33 e Å^−^^3^
2509 reflections	Extinction correction: none
154 parameters	

### Fractional atomic coordinates and isotropic or equivalent isotropic displacement parameters (Å^2^)

**Table:**

	*x*	*y*	*z*	*U*_iso_*/*U*_eq_	
S1	0.72343 (3)	0.06408 (5)	0.19253 (3)	0.0234	
C2	0.79732 (14)	−0.11421 (19)	0.19970 (14)	0.0244	
C3	0.90751 (14)	−0.0827 (2)	0.19854 (14)	0.0252	
C4	0.90283 (13)	−0.0099 (2)	0.08935 (14)	0.0244	
N5	0.84522 (11)	0.13793 (17)	0.06888 (12)	0.0225	
C6	0.76970 (12)	0.17936 (19)	0.10687 (13)	0.0199	
N7	0.72435 (11)	0.31807 (16)	0.08222 (11)	0.0217	
C8	0.65460 (13)	0.37922 (18)	0.13689 (14)	0.0210	
C9	0.69975 (13)	0.4718 (2)	0.23077 (14)	0.0224	
C10	0.63438 (14)	0.5346 (2)	0.28445 (15)	0.0278	
C11	0.52719 (15)	0.5046 (2)	0.24466 (17)	0.0319	
C12	0.48404 (14)	0.4128 (2)	0.15214 (17)	0.0300	
C13	0.54694 (13)	0.3482 (2)	0.09525 (15)	0.0255	
C14	0.49879 (15)	0.2481 (2)	−0.00495 (16)	0.0319	
C15	0.81647 (13)	0.4980 (2)	0.27462 (14)	0.0249	
Cl16	0.18401 (3)	0.10294 (5)	0.51765 (3)	0.0238	
O17	0.94136 (9)	0.31268 (14)	−0.05720 (10)	0.0272	
H21	0.8012	−0.1644	0.2678	0.0280*	
H31	0.9450	−0.1803	0.2067	0.0276*	
H32	0.9432	−0.0145	0.2573	0.0277*	
H41	0.9748	0.0101	0.0908	0.0289*	
H42	0.8694	−0.0804	0.0300	0.0289*	
H141	0.4312	0.2885	−0.0488	0.0475*	
H142	0.5426	0.2414	−0.0510	0.0467*	
H143	0.4889	0.1450	0.0189	0.0475*	
H151	0.8332	0.5724	0.3339	0.0357*	
H152	0.8415	0.5357	0.2174	0.0356*	
H153	0.8515	0.4017	0.3030	0.0359*	
H171	0.9124	0.3966	−0.0570	0.0391*	
H172	1.0056	0.3286	−0.0335	0.0395*	
H22	0.7604	−0.1794	0.1379	0.0278*	
H5	0.8667	0.2066	0.0318	0.0267*	
H7	0.7466	0.3812	0.0417	0.0250*	
H10	0.6635	0.5960	0.3474	0.0320*	
H11	0.4838	0.5483	0.2825	0.0362*	
H12	0.4111	0.3933	0.1255	0.0341*	

### Atomic displacement parameters (Å^2^)

**Table:**

	*U*^11^	*U*^22^	*U*^33^	*U*^12^	*U*^13^	*U*^23^
S1	0.0236 (2)	0.0216 (2)	0.0306 (2)	0.00450 (15)	0.01642 (17)	0.00271 (14)
C2	0.0292 (9)	0.0208 (8)	0.0269 (8)	0.0023 (6)	0.0143 (7)	0.0055 (6)
C3	0.0257 (8)	0.0265 (8)	0.0251 (8)	−0.0001 (7)	0.0109 (7)	0.0059 (7)
C4	0.0227 (8)	0.0262 (9)	0.0274 (8)	−0.0012 (7)	0.0127 (7)	0.0037 (6)
N5	0.0227 (7)	0.0222 (7)	0.0268 (7)	0.0015 (5)	0.0137 (5)	0.0004 (5)
C6	0.0186 (7)	0.0218 (8)	0.0203 (7)	−0.0012 (6)	0.0076 (6)	−0.0019 (6)
N7	0.0237 (7)	0.0206 (7)	0.0245 (7)	0.0018 (5)	0.0130 (6)	0.0002 (5)
C8	0.0203 (7)	0.0193 (8)	0.0258 (8)	0.0046 (6)	0.0107 (6)	0.0032 (6)
C9	0.0225 (8)	0.0204 (8)	0.0258 (8)	0.0035 (6)	0.0103 (7)	0.0035 (6)
C10	0.0297 (9)	0.0274 (8)	0.0296 (9)	−0.0003 (7)	0.0140 (7)	0.0043 (7)
C11	0.0274 (9)	0.0319 (10)	0.0432 (10)	0.0024 (8)	0.0209 (8)	0.0065 (7)
C12	0.0181 (8)	0.0290 (9)	0.0442 (11)	0.0072 (8)	0.0121 (7)	0.0020 (7)
C13	0.0221 (8)	0.0218 (8)	0.0319 (9)	0.0052 (7)	0.0078 (7)	0.0008 (6)
C14	0.0264 (8)	0.0263 (9)	0.0382 (10)	0.0001 (8)	0.0042 (7)	−0.0029 (7)
C15	0.0224 (8)	0.0254 (9)	0.0259 (8)	0.0012 (7)	0.0065 (7)	0.0007 (6)
Cl16	0.0232 (2)	0.0239 (2)	0.0266 (2)	0.00033 (14)	0.01150 (16)	0.00127 (14)
O17	0.0244 (6)	0.0248 (6)	0.0351 (7)	−0.0025 (5)	0.0135 (5)	−0.0028 (5)

### Geometric parameters (Å, °)

**Table:**

S1—C2	1.8215 (17)	C9—C10	1.391 (2)
S1—C6	1.7403 (16)	C9—C15	1.501 (2)
C2—C3	1.512 (2)	C10—C11	1.387 (3)
C2—H21	0.959	C10—H10	0.936
C2—H22	0.964	C11—C12	1.383 (3)
C3—C4	1.513 (2)	C11—H11	0.950
C3—H31	0.971	C12—C13	1.401 (3)
C3—H32	0.952	C12—H12	0.942
C4—N5	1.474 (2)	C13—C14	1.504 (3)
C4—H41	0.977	C14—H141	0.964
C4—H42	0.961	C14—H142	0.961
N5—C6	1.312 (2)	C14—H143	0.966
N5—H5	0.866	C15—H151	0.963
C6—N7	1.336 (2)	C15—H152	0.957
N7—C8	1.442 (2)	C15—H153	0.967
N7—H7	0.870	O17—H171	0.825
C8—C9	1.404 (2)	O17—H172	0.829
C8—C13	1.395 (2)		
			
C2—S1—C6	102.42 (8)	C9—C8—C13	122.61 (15)
S1—C2—C3	111.57 (12)	C8—C9—C10	118.55 (16)
S1—C2—H21	107.2	C8—C9—C15	120.71 (15)
C3—C2—H21	109.3	C10—C9—C15	120.71 (16)
S1—C2—H22	109.2	C9—C10—C11	119.76 (17)
C3—C2—H22	109.9	C9—C10—H10	119.4
H21—C2—H22	109.7	C11—C10—H10	120.8
C2—C3—C4	109.91 (14)	C10—C11—C12	120.93 (16)
C2—C3—H31	108.6	C10—C11—H11	118.6
C4—C3—H31	108.9	C12—C11—H11	120.5
C2—C3—H32	110.3	C11—C12—C13	121.14 (16)
C4—C3—H32	109.0	C11—C12—H12	120.4
H31—C3—H32	110.2	C13—C12—H12	118.5
C3—C4—N5	112.65 (13)	C12—C13—C8	117.01 (16)
C3—C4—H41	108.4	C12—C13—C14	120.45 (16)
N5—C4—H41	108.0	C8—C13—C14	122.53 (16)
C3—C4—H42	109.2	C13—C14—H141	110.2
N5—C4—H42	109.1	C13—C14—H142	112.1
H41—C4—H42	109.3	H141—C14—H142	108.5
C4—N5—C6	126.70 (14)	C13—C14—H143	109.2
C4—N5—H5	116.2	H141—C14—H143	108.5
C6—N5—H5	116.9	H142—C14—H143	108.3
S1—C6—N5	123.83 (13)	C9—C15—H151	109.9
S1—C6—N7	115.66 (12)	C9—C15—H152	110.8
N5—C6—N7	120.50 (15)	H151—C15—H152	108.8
C6—N7—C8	122.35 (13)	C9—C15—H153	109.3
C6—N7—H7	118.9	H151—C15—H153	108.8
C8—N7—H7	117.6	H152—C15—H153	109.3
N7—C8—C9	117.10 (14)	H171—O17—H172	106.9
N7—C8—C13	120.28 (15)		

### Hydrogen-bond geometry (Å, °)

**Table:**

*D*—H···*A*	*D*—H	H···*A*	*D*···*A*	*D*—H···*A*
N5—H5···O17	0.87	1.97	2.815 (2)	163
O17—H171···Cl16^i^	0.82	2.36	3.158 (1)	164
N7—H7···Cl16^i^	0.87	2.37	3.204 (1)	162
O17—H172···Cl16^ii^	0.83	2.35	3.171 (1)	173

Symmetry codes: (i) −*x*+1, *y*+1/2, −*z*+1/2; (ii) *x*+1, −*y*+1/2, *z*−1/2.
